# Patient-reported outcome measures: selection of a valid questionnaire for routine symptom assessment in patients with advanced chronic kidney disease – a four-phase mixed methods study

**DOI:** 10.1186/s12882-019-1521-9

**Published:** 2019-09-02

**Authors:** Esmee M. van der Willik, Yvette Meuleman, Karen Prantl, Giel van Rijn, Willem Jan W. Bos, Frans J. van Ittersum, Hans A. J. Bart, Marc H. Hemmelder, Friedo W. Dekker

**Affiliations:** 1 Department of Clinical Epidemiology, Leiden University Medical Centre, PO Box 9600, 2300 RC Leiden, The Netherlands; 2 Dutch Kidney Patients Association, Groot Hertoginnelaan 34, 1405 EE, Bussum, The Netherlands; 3 Department of Nephrology, Leiden University Medical Centre, PO Box 9600, 2300 RC Leiden, The Netherlands; 4 Department of Internal Medicine, St Antonius Hospital, Koekoekslaan 1, 3435 CM Nieuwegein, The Netherlands; 5 Department of Nephrology, Amsterdam University Medical Centre, De Boelelaan 1117, 1081 HV Amsterdam, The Netherlands; 6 Nefrovisie Foundation, Moreelsepark 1, 3511 EP Utrecht, The Netherlands; 7 Department of Internal Medicine, Medical Centre Leeuwarden, Henri Dunantweg 2, 8934 AD Leeuwarden, The Netherlands

**Keywords:** Chronic kidney disease (CKD), End-stage kidney disease (ESKD), Pre-dialysis, Dialysis, Symptom burden, Questionnaire, Patient-reported outcome measures (PROMs), Value-based healthcare

## Abstract

**Background:**

Patient-reported outcome measures (PROMs) are becoming increasingly important in healthcare. In nephrology, there is no agreement on which chronic kidney disease (CKD) symptom questionnaire to use. Therefore, the aim of this study is to select a valid symptom questionnaire for routine assessment in patients with advanced CKD.

**Methods:**

A four-phase mixed methods approach, using qualitative and quantitative research methods, was applied. First, a systematic literature search was conducted to retrieve existing symptom questionnaires. Second, a symptom list was created including all symptoms in existing questionnaires and symptoms mentioned in interviews with patients with CKD, from which symptom clusters were identified. Next, questionnaires were selected based on predefined criteria regarding content validity. Last, two online feedback panels of patients with CKD (*n* = 151) and experts (*n* = 6) reviewed the most promising questionnaires.

**Results:**

The literature search identified 121 questionnaires, of which 28 were potentially suitable for symptom assessment in patients with advanced CKD. 101 unique symptoms and 10 symptom clusters were distinguished. Based on predefined criteria, the Dialysis Symptom Index (DSI) and Palliative Care Outcome Scale-Renal Version (IPOS-Renal) were selected and reviewed by feedback panels. Patients needed 5.4 and 7.5 min to complete the DSI and IPOS-Renal, respectively (*p* < 0.001). Patients experienced the DSI as more specific, complete and straightforward compared to the IPOS-Renal.

**Conclusions:**

The DSI was found to be valid and reliable, the most relevant, complete, and comprehensible symptom questionnaire available for routine assessment in patients with advanced CKD. Routine PROMs collection could be of great value to healthcare, both at individual patient and national level. Feedback on scores and involvement of healthcare providers may promote adaptation and implementation in healthcare.

**Electronic supplementary material:**

The online version of this article (10.1186/s12882-019-1521-9) contains supplementary material, which is available to authorized users.

## Background

The last decade there has been a shift in focus towards a more patient-centred and value-based healthcare. As described by Michael E. Porter, value in healthcare depends on the outcomes achieved and should be defined around the patient [1]. With this change, patient-reported outcomes (PROs) are becoming increasingly important in healthcare [1–4]. PRO measures (PROMs) can be used to quantify a wide variety of concepts of health that are relevant to the patient, such as quality of life, functional status and symptom burden [2, 5].

Until recently, PROMs were mainly used in research settings. However, PROMs are increasingly being applied for clinical management in individual patients and evaluation of quality of care [4, 6]. PROMs may enhance understanding of patients’ symptoms and needs, and have the potential to improve patient outcomes and engagement in decision making [4, 7, 8]. The use of PROMs is nowadays recommended to be implemented and routinely used in clinical practice [4, 9, 10].

Broadly a PROM can be classified as a generic or disease specific instrument. Generic PROMs measure general aspects of patients’ health status, such as functional status or quality of life. Disease specific PROMs are tailored to a specific condition and address aspects of disease experience and symptoms, making these PROMs in general more sensitive and responsive to change in disease burden [2, 4, 5, 10]. Often, both generic and disease specific PROMs are used to enable comparisons across and within populations [4, 5, 10].

Also in nephrology, routine collection of PROMs can be of added value. [11] Patients with advanced chronic kidney disease (CKD) experience a poor health-related quality of life (HRQOL) and numerous physical and emotional disease related symptoms [12–14]. Moreover, in patients with advanced CKD, HRQOL levels generally decrease and symptom burden generally increases as the disease progresses [15]. Despite their relevance, many symptoms in patients with advanced CKD remain unnoticed. This may be partly explained by patients being reluctant to share their experienced symptoms, particularly due to feelings of guilt about wasting clinicians’ or other patients’ time [16]. Additionally, clinicians frequently are not able to identify the full spectrum of experienced symptoms and their severity, resulting in under-recognition and under-treatment of symptoms [14, 17–19]. Routine symptom assessment, using a questionnaire that fits patients’ needs, could provide insight and guidance for symptom management [16, 20]. Symptom management has been identified as top priority by patients with advanced CKD [21].

Although the relevance of patients’ perspective is recognized, PROMs have not yet been widely implemented in nephrology [2, 9, 11]. Currently, methods and instruments needed for implementation of PROMs in patients with advanced CKD, including patients with end-stage kidney disease (ESKD) with and without dialysis, are being explored in the Netherlands. Some generic health questionnaires are considered to be appropriate instruments for this purpose [9, 22]. However, there is no agreement on which questionnaire is most suitable to measure the broad spectrum of symptoms that patients with advanced CKD experience [9, 23]. Therefore, the aim of this study is to systematically select the most suitable CKD-specific symptom questionnaire for routine assessment in patients with advanced CKD and ESKD using a four-phase mixed methods approach.

## Methods

### Overview

This study is part of the development of a national registry of PROMs, which will be included in the Dutch Renal Registry (Renine) [www.nefrovisie.nl/nefrodata]. For now, the PROMs registry is primarily aimed at patients with advanced CKD, including patients with ESKD receiving dialysis or without renal replacement therapy (RRT). Patients will be followed over time across different stages and treatments (e.g. advanced CKD, ESKD, with and without RRT), and therefore, we have chosen not to restrict this study to subpopulations, but to focus on CKD in general, taking all existing CKD-specific symptom questionnaires into consideration.

In this study, the focus was on the content validity of the symptom questionnaire, defined as “the relevance, comprehensiveness, and comprehensibility of the PROM for the construct, target population, and context of use of interest” [24]. According to the COnsensus-based Standards for the selection of health Measurement INstruments (COSMIN) standards, content validity is the most important and first to be considered measurement property in selecting a PROM [24, 25]. Furthermore, since numerous symptom questionnaires are already available [2, 26], it would be preferable to select an existing questionnaire instead of developing a new one. As an alternative for organizing focus groups and interviews with patients to identify domains of symptoms relevant to patients, we searched and used existing CKD symptom questionnaires, assuming that they all have attempted to include the most important domains and items. By combining all these questionnaires, we make use of a much wider variation in patients, methods, clinical settings and countries to gather content-wise relevant domains for CKD.

A four-phase approach, combining qualitative and quantitative research methods, was applied: 1) conduct a systematic literature search to retrieve all existing symptom questionnaires used in patients with CKD. 2) Create a complete list of unique symptoms from all symptom questionnaires and interviews with patients with advanced CKD. Cluster these symptoms into relevant symptom groups. 3) Select symptom questionnaires based on criteria to ensure content validity, including the completeness, relevance and comprehensibility for the advanced CKD population and context of routine care [24]. 4) Evaluate the most promising symptom questionnaires using a panel of patients with advanced CKD and experts (i.e. experienced questionnaire assessors). Below the four phases are described in detail.

### Systematic literature search - phase 1

A systematic literature search was performed to identify all existing symptom questionnaires developed and/or used in patients with CKD. A query was constructed using numerous synonyms or identifiers for the keywords ‘chronic kidney disease’, ‘symptoms’ and ‘questionnaires’ (Additional file 1). The search was restricted to studies published in the English or Dutch language. Studies conducted in individuals < 18 years of age were excluded.

The search was executed in PubMed by two independent reviewers (EvdW and GvR). Titles were screened and found to be eligible when describing one or more symptoms or the use of a symptom questionnaire in patients with CKD. Next, the abstracts of articles included by at least one of the reviewers were screened to identify existing symptom questionnaires. Systematic reviews describing the use of questionnaires in patients with CKD were screened full text to make sure that all existing symptom questionnaires were included.

We aim to select a symptom questionnaire addressing the full range of symptoms experienced by the total CKD population. To distinguish such broad symptom questionnaires from in-depth questionnaires addressing only one or two specific symptoms (e.g. depression or fatigue questionnaires), we excluded symptom questionnaires addressing less than four physical or emotional symptoms [26]. Additionally, questionnaires focussing only on transplant-specific symptoms and generic health questionnaires (e.g. HRQOL or activities of daily living questionnaires) were excluded.

### Symptom list and clustering - phase 2

#### Symptoms from questionnaires

A list of symptoms was created from all symptoms included in the questionnaires. To collect only unique symptoms, overlapping symptoms were combined (e.g. ‘Tingling in feet or hands’ as a combination of ‘Tingling in feet’ and ‘Tingling in hands’).

#### Analysis of videotaped interviews

To assure completeness of the symptom list, 18 videotaped interviews with patients with advanced CKD were analysed to check for missing symptoms. Patients received haemodialysis (*n* = 13), peritoneal dialysis (*n* = 3) or no RRT (*n* = 2), were 20–83 years old, and half of them was male. The interviews were conducted by two experienced male interviewers (HB and FvdZ), who were not involved in the patients’ treatment. The videos were obtained from the Dutch Kidney Patients Association (NVN) [27] and were developed to inform and support patients with CKD in making future choices regarding therapy. During the semi-structured interviews, different aspects of living with CKD were discussed, including aspects about disease, treatment, physical functioning, psychosocial aspects, relationships and quality of life. As a result of patient’s answers, additional themes were sometimes introduced including symptoms that patients experience and considered relevant. The NVN and the interviewed patients gave permission to use this material for this research purposes. The videotaped interviews were watched and analysed by two independent researchers (GvR and EvdW). All symptoms mentioned by patients were written down verbatim and subsequently compared to the symptom list derived from the questionnaires (phase 1). Symptoms that were not yet on the list were added.

#### Clustering of symptoms

The total list of unique symptoms was divided into clusters to identify themes that describe the broad spectrum of symptoms experienced by patients with CKD. Clustering was done by two independent healthcare professionals: a nephrologist (JR) and a nurse practitioner (NBB) specialized in pre-dialysis and dialysis care, both experienced in clinical practice and research. JR and NNB discussed the symptoms and identified clusters inductively by constant comparison and grouping of similar type of symptoms. Clusters and corresponding symptoms were discussed until consensus was reached.

### Preliminary selection of symptom questionnaire - phase 3

A set of criteria (Table 1) was applied to make a preliminary selection of symptom questionnaires that are relevant, complete and comprehensible for patients with advanced CKD or ESKD in routine care setting.

**Table 1:** Criteria for symptom questionnaires suitable for routine assessment in patients with advanced chronic kidney disease (CKD)

Criterion	Description
A. Symptom clusters	≥ 90% cluster coverage The variety of symptoms experienced in CKD requires a questionnaire addressing a wide range of symptoms. Preferably all, but at least 90% of the clusters should be covered by the questionnaire.
B. Questionnaire length	≤ 90 items The questionnaire needs to have an appropriate length to be suitable for routine assessment. The questionnaires should have a maximum length of 15 min to complete [28], which we expect to be exceeded by a questionnaire addressing ≥90 items [29].
C. Applicable to advanced CKD population	Developed and validated in advanced CKD The questionnaires should be applicable to the advanced CKD population. Preference is given to a questionnaire both developed and validated in patients with advanced CKD.
D. Suitable for use in routine care	Straightforward and clear For a questionnaire addressing more than symptoms only, the symptoms need to be concentrated together (i.e. symptom questions are not mixed with other questions), so that a separate and valid symptom questionnaire can be extracted. Since patient’s ability to concentrate and understand difficult items may be impaired, the questionnaire needs to be straightforward with appropriate and easy to interpret items and scales [29].

### Feedback panels - phase 4

Dutch versions of the most promising questionnaires were evaluated by two online feedback panels facilitated by the NVN. One panel consisted of 151 patients receiving different treatments: pre-dialysis (CKD stage 4-5), haemodialysis, peritoneal dialysis and transplantation. The patients in this panel were randomly assigned to one of the selected questionnaires. Patients assessed only one questionnaire so that their judgement on the assigned questionnaire was based on their personal opinion, experiences and needs, and not influenced by the content or structure of another questionnaire. The second panel consisted of six experienced questionnaire assessors, namely NVN patient representatives who advise on research (e.g. questionnaire development). Five of these experts were CKD patient and one person was a relative of a CKD patient. To enable a direct comparison of the questionnaires, this panel of experts compared all questionnaires from the previous phase.

To review the questionnaire, patients were asked to complete the questionnaire and to answer additional questions. Questions concerned the content and structure of the questionnaire, including: time needed for completion, burden of completing the questionnaire, desired frequency of questionnaire assessment, unclear questions, unnecessary questions, missing questions with room to report three additional symptoms, and other suggestions or comments. The time to complete the questionnaire was measured electronically (i.e. objective time). Patients also estimated the time to complete the questionnaire, hereafter referred to as subjective time. Differences between the questionnaires in objective and subjective time to complete were presented as geometric mean.

Statistical analyses were performed using SPSS V.23.0. *P* < 0.05 was considered statistically significant. To evaluate differences in patient, treatment and questionnaire characteristics, Student’s t-test and Chi-square tests of association were performed. To test the reliability of the symptom burden score, Cronbach’s alpha coefficients were calculated. A sensitivity analysis using one-way ANOVA and Chi-square tests was conducted to determine if the results from the patient panel are the same for transplant patients compared to patients on dialysis or without RRT.

## Results

### Systematic literature search - phase 1

**Fig. 1:**
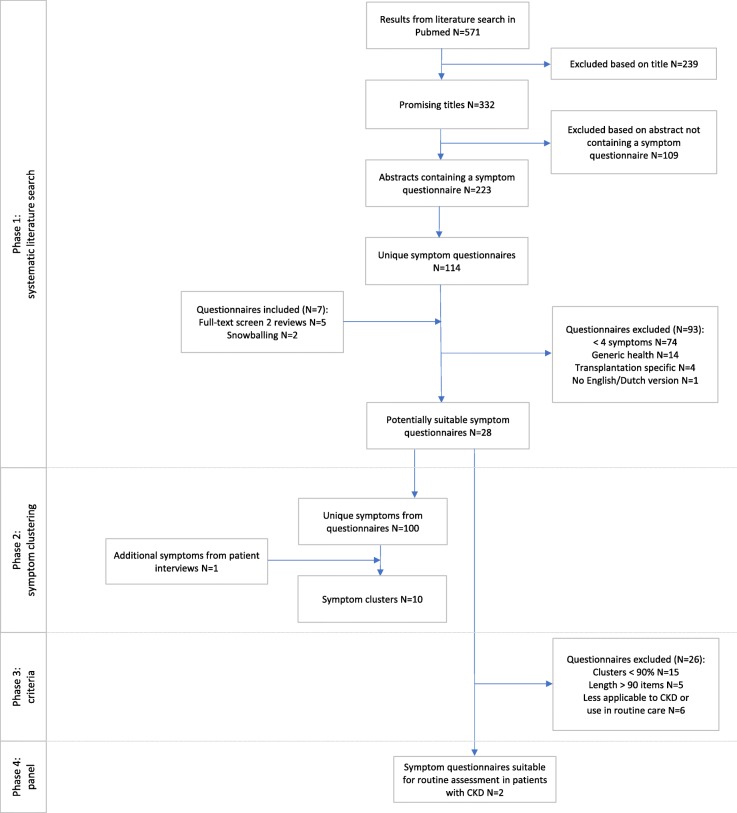
Flow chart of the selection of a valid CKD-specific symptom questionnaire

Figure 1 shows a flow diagram of the literature search and questionnaire selection. The search strategy identified 571 articles, of which 223 articles were included based on title and abstract. From these articles, including two full text reviews, and through snowballing, 121 unique symptom questionnaires were identified. Of these questionnaires, 93 were excluded (mainly because less than four symptoms were addressed, see Fig. 1), resulting in 28 symptom questionnaires for further investigation.

### Symptom list and clustering - phase 2

A complete symptom list was created from the 28 symptom questionnaires. One hundred unique symptoms were identified from these questionnaires. Analysis of the videotaped interviews with patients with advanced CKD resulted in one additional symptom (Additional file 2: Table S2). From this symptom list, two healthcare professionals distinguished the following ten clusters: general symptoms, night’s rest, gastroenterology, cardiopulmonary, central nervous system, musculoskeletal, skin, head/throat, psychosocial and sex. The total symptom list categorized into ten clusters is available in Additional file 2: Table S2.

### Preliminary selection of symptom questionnaire - phase 3

In the third phase, the previous two steps were combined: the 28 symptom questionnaires were judged on their coverage of at least nine out of ten symptom clusters. Fifteen questionnaires were excluded based on this criterion (criterion A). An additional three questionnaires were excluded due to their extensive length (criterion B), leaving ten questionnaires for further examination.

Table 2 shows the characteristics of the ten questionnaires on which the questionnaires were compared and evaluated. Six out of ten questionnaires were both developed and validated in an advanced CKD population, meeting criterion C. Two of these six are derivatives of a third questionnaire; the two questionnaires include exactly the same symptoms but also distinguish how much a symptom bothers, the severity and the frequency of symptoms and hereby exceed the determined maximum length (criterion B). Another two questionnaires address a broader perspective than symptoms only. The questions regarding symptoms are spread across the questionnaire, which does not satisfy criterion D. Based on the criteria two questionnaires were selected for further consideration in the next phase.

**Table 2:** Characteristics of 10 suitable symptom questionnaires for routine assessment in patients with advanced CKD based on cluster coverage and suitable length

	CHEQ	CKD-SBI^Δ^	Curtin	DFSSBI^Δ^	DSI^Δ^	IPOS-Renal	KDQOL-SF	MSAS	MSAS-SF/+renal symptoms	RSCL
Development population	Dialysis	CKD 4–5	Dialysis	Dialysis	Dialysis	CKD 4–5	Dialysis	*Cancer*	*Cancer*/CKD 5	*Cancer*
Validated for advanced CKD	Yes	Yes	*No*	Yes	Yes	Yes	Yes	*No*	Yes/*No*	*No*
Total number of items	80	33*	47	31*	30	21	82	33*	33/39	39
Separate symptom component	*No*	Yes	Yes	Yes	Yes	Yes	*No*	Yes	Yes	Yes
Number of items per cluster										
General symptoms	3	2	4	2	2	2	6	5	5	3
Night’s rest	3	2	4	2	2	2	4	2	2	1
Gastroenterology	4	5	8	5	5	5	2	7	7	7
Cardiopulmonary	2	5	3	4	4	1	3	3	3/4	1
Central nervous system	3	4	7	4	4	1	3	3	3/5	4
Musculoskeletal	1	3	5	3	3	1	2	0	0/3	2
Skin	2	2	3	2	2	2	2	4	4/5	1
Head and throat	1	1	4	1	1	1	0	3	3	3
Psychosocial	12	6	6	6	5	4	12	4	4	7
Sex	3	2	3	2	2	0	2	1	1	1
Open questions	1	1	0	0	0	2	0	1	1	0
Burden rating scale	2 till 7-point scale	11-point Likert scale	5-point Likert scale	5- and 10-point scale	5-point Likert scale	5-point Likert scale	2 till 10-point scale	4- and 5-point scale	4- and 5-point scale	4-point scale
Recall^#^	4 weeks/3 months/in general	4 weeks	4 weeks	1–3 days^	1 week	1 week	4 weeks/in general	1 week	1 week	1 week

*Italic* marks reasons for exclusion

^Δ^ CKD-SBI and DFSSBI are derivatives of the DSI and include the same symptoms (with 1 or 2 additional symptom(s))

*For each item, the patient was expected to report the frequency, severity and bothersome

#The time period addressed, e.g. the recall period is 1 week for the question “did you experience this symptom during the past week?”

^The time period was defined as “since your last dialysis treatment” and thus depended on the frequency of dialysis treatment, which was estimated at 3–7 times per week

Abbreviations and questionnaires: CKD, Chronic Kidney Disease; CHEQ, CHOICE Health Experience Questionnaire [30]; CKD-SBI, CKD Symptom Burden Index [31]; Curtin [32]; DFSSBI, Dialysis Frequency, Severity, and Symptom Burden Index [33]; DSI, Dialysis Symptom Index [34]; IPOS-Renal, Palliative Care Outcome Scale - Renal Version [35]; KDQOL-SF, Kidney Disease Quality of Life instrument - Short Form [36]; MSAS, Memorial Symptom Assessment Scale [37]; MSAS-SF, Memorial Symptom Assessment Scale-Short Form [38]; MSAS-SF with additional renal symptoms [39]; RSCL, Rotterdam Symptom Checklist [40]

### Feedback panels - phase 4

#### Feedback panels

The Dialysis Symptom Index (DSI) [34] and Palliative Care Outcome Scale – Renal Version (IPOS-Renal) [35] were judged by two online panels of patients and experts. Patients were randomly assigned to a questionnaire (Table 3). In total 127 patients (84.1%) received RRT, of which 27 dialysis (17.9%) and 100 transplantation (66.2%). The second panel of six experts evaluated and compared both questionnaires.

**Table 3:** Comparison of two CKD-specific symptom questionnaires based on feedback of the patient panel (*n* = 151)

	DSI (*n* = 76)	IPOS-Renal (*n* = 75)	*p*-value
Age (years)	60.6 (12.5)	60.2 (10.4)	0.8
Treatment modality			0.5
Pre-dialysis	6 (7.9)	13 (17.3)	
Haemodialysis	8 (10.5)	9 (12.0)	
Peritoneal dialysis	6 (7.9)	4 (5.3)	
Transplant	53 (69.7)	47 (62.7)	
Other	3 (3.9)	2 (2.7)	
Objective time to complete* (minutes)	5.4 (1.6)	7.5 (1.8)	< 0.001
Subjective time to complete* (minutes)	3.2 (1.8)	4.8 (1.6)	< 0.001
Number of symptoms reported^	12.0 (6.5)	8.0 (4.1)	< 0.001
Additional 1–3 symptoms reported^#^	21 (27.6)	25 (33.3)	0.5
Burdensome of questionnaire (yes)	4 (5.3)	2 (2.7)	0.4
Appropriate frequency of submission (times per year)	2.7 (1.8)	2.9 (2.2)	0.6

Values are shown in *n* (%) or mean (SD)

The DSI and IPOS-Renal questionnaires showed good reliability for symptom burden score with Cronbach’s alpha values of 0.90 and 0.86, respectively

*Objective time to complete was defined as the difference in minutes between the start and completion of the online questionnaire. Subjective time to complete is the time to complete estimated by the patient. Values shown as geometric mean (SD)

^The number of symptoms reported is based on the symptoms defined in the questionnaire and rated by the patient as bothering a little bit to very much (or affecting slightly to overwhelmingly)

^#^The number of patients reporting an additional 1 to 3 symptoms not mentioned in de questionnaire

Abbreviations: CKD, Chronic Kidney Disease; DSI, Dialysis Symptom Index; IPOS-Renal, Palliative Care Outcome Scale - Renal Version

#### Time to complete

The patient panel needed on average (standard deviation; SD) 5.4 (1.6) minutes to complete the DSI and 7.5 (1.8) minutes to complete the IPOS-Renal (*p* < 0.001). Also subjectively the DSI was less time consuming than the IPOS-Renal, with a difference in geometric mean of 1.6 min (*p* < 0.001). The time to complete estimated by experts ranged from 2 to 15 and 3 to 20 minutes for the DSI and IPOS-Renal, respectively.

#### Burden and frequency

Four and two patients of the online patient panel experienced, respectively, the DSI and IPOS-renal as burdensome. All experts indicated that both questionnaires were not burdensome. For both questionnaires, most patients prefer to complete the questionnaire two or four times per year. Most experts (4 out of 6) desired four times per year. In both panels, participants noted that the questionnaire should be filled in prior to each consultation with the nephrologist.

#### Questions

Both panels indicated that, overall, the questions in both questionnaires were clear. All experts fully agreed that both questionnaires were easy to interpret and one expert added that the questionnaires were also comprehensible for patients with low literacy. For the IPOS-Renal some patients and one expert noted that the questions might be too generally formulated, which can cause confusion or difficulties to interpret a question. Also, some patients indicated that some questions might not be applicable to all patients or treatment modalities. For the DSI some patients mentioned that questions about sexual problems may be not applicable to all patients. Two experts indicated that, in comparison to the DSI, some symptoms may be missed when using the IPOS-Renal, but these experts did not mention which symptoms were lacking. For both questionnaires patients reported additional symptoms, which were all covered by the defined clusters. Patients reported more symptoms using the DSI than using the IPOS-Renal, with 12.0 and 8.0 experienced symptoms, respectively (*p* < 0.001) (Table 3).

#### Comments

Patients made comments similar to the answers described above (see Questions). About the DSI several patients reported that they experienced the questionnaire as pleasant, clear and enlightening. For both questionnaires patients suggested to add questions on treatment and “how patients experience their lives”. Additionally, the patients pointed out that feedback on their results and involvement of the nephrologist are highly important.

#### Preference

The experts compared both questionnaires. Five out of six experts preferred the DSI. They qualified the DSI as more specific and complete, and believed that the questions were more clear and easier to fill in than the IPOS-Renal. Two experts, however, also mentioned that the lay-out of the IPOS-Renal was visually more attractive than the DSI.

#### Sensitivity analysis

Results of the patient panel stratified for transplant and non-transplant patients are available in Additional file 3: Table S3. Similar differences between the DSI and IPOS-Renal were found in transplant and non-transplant patients. Transplant patients completed both questionnaires faster (*p* = n.s.) and reported less symptoms (*p* = n.s.) compared to non-transplant patients. However, both transplant and non-transplant patients needed less time to complete the DSI (*p* < 0.05) and reported more symptoms using the DSI compared to the IPOS-Renal (*p* < 0.05). Also comments regarding the content and structure of the questionnaires were similar in transplant and non-transplant patients.

## Discussion

The aim of this study was to select a valid CKD specific symptom questionnaire for routine assessment in patients with advanced CKD or ESKD. The first two phases, the literature search and symptom clustering, resulted in 28 potentially suitable symptom questionnaires and ten symptom clusters. During the third phase, two questionnaires were selected based on their relevance, completeness and comprehensibility to routine assessment in patients with CKD: the DSI and IPOS-Renal. These two questionnaires were reviewed by panels of patients and experts in the fourth phase. The results of the panel reviews showed that the DSI was the most complete, specific and comprehensible symptom questionnaire. Therefore, the DSI was considered to be the most suitable symptom questionnaire currently available for routine assessment in patients with advanced CKD or ESKD.

Previous literature and current findings support the completeness and straightforwardness of the DSI. First, the patient panel reported 12 symptoms using the DSI, which is 1.5 times the number of symptoms reported when using the IPOS-Renal. We believe that this increased score is due to differences in completeness of the questionnaires rather than differences in characteristics between patients. Similar numbers of symptoms are also presented in previous literature [41]. Furthermore, a recent study showed that symptoms of insomnia, fatigue, cramping, anxiety, depression and frustration were considered top-priority by dialysis patients. Such physical and emotional symptoms are also included in the DSI [42]. Still, additional symptoms were mentioned by the patients assessing the DSI. Therefore, we propose to retain the possibility to report additional symptoms as this may favour the completeness and patient satisfaction [43]. Besides this, the time to complete the questionnaire reflects the straightforwardness of the DSI. Although the DSI contains more items than the IPOS-Renal, patients needed less time to complete the questionnaire. This might suggest that the DSI is more clear and easier to complete for patients.

We believe that routine symptom assessment can contribute to a more patient-centred healthcare system and improvement in quality of care. Routine assessment enables patients and healthcare professionals to track changes in symptom burden over time, which may result in a more complete and better understanding of patients’ symptoms and needs. Routine assessment may also yield valuable information for the evaluation of effectiveness of treatment and the progression of symptoms.

Herein, the provision of feedback on PROM score to patients and healthcare providers, both on individual and on aggregated level, may be of great importance [44]. At the individual patient level, feedback may enhance communication between patients and healthcare professionals, which is considered highly important by patients with advanced CKD [21]. Moreover, results of similar patients could provide insight in what to expect in the future and may promote patient engagement in decision making [3, 45]. Additional to the provision of feedback, the involvement of clinicians was considered very important by several patients and is expected to contribute to a successful implementation and a more patient-centred healthcare [46].

At centre or national level, performance and variation in outcomes between centres can be mapped out and may promote initiatives to improve quality of care. Besides, patient outcomes are of great value to the already available clinical performance measures, which mainly consider structure and process of care [47, 48]. So far, PROMs have been mainly used in scientific research and less often for nationwide assessment in clinical practice [4, 9]. Consequently, little is known about how PROMs can be best deployed to achieve quality improvement [49, 50].

Further research is needed to investigate how PROMs can be best used in clinical practice to improve symptom management, shared decision making and to address patients’ needs. We propose to assess and discuss symptoms using the DSI twice per year, in order to gain insight into symptom development with a minimal burden to patients and to healthcare professionals. In addition to a suitable questionnaire, successful implementation of PROMs into routine care requires planning, facilities (e.g. electronic system to collect and report PROM scores) and involvement of all stakeholders (e.g. patients, healthcare professionals and researchers) [51]. Furthermore, barriers may be encountered when implementing PROMs into routine care, including low response rates, organizational struggles or low commitment from patients or healthcare professionals [11]. To facilitate implementation and sustainability, it is vital to take these barriers into account, by, for example, providing information and communication systems to adequately collect data and discuss PROM-scores. We suggest to test the PROMs in collaboration with all stakeholders so that it fits the workflow and priorities in routine care.

A unique feature of this study is the four-phase mixed methods approach with both qualitative and quantitative research methods. Especially with the combination of these methods we believe to have selected a valid and reliable symptom questionnaire that is relevant, complete and appropriate for the population and context of interest. First, this method addressed all criteria for evaluation of content validity as established in the COSMIN standard [24]. Second, with the use of all existing symptom questionnaires, we believe to have reached completeness and to have identified the domains that are most relevant to the patient, more so than would be possible when conducting a single study. This conclusion is also supported by patients’ input: the analysis of the interviews with patients with CKD resulted in only one additional symptom and no new symptoms or domains were mentioned by the patient panel. Third, patients, healthcare professionals and experts were involved in this study. Particularly patient involvement was considered highly important, because patients’ perspective helps to select the questionnaire that is most complete, comprehensible and relevant to them. This might increase the probability of completing the questionnaire when implemented in daily practice [52]. By using this mixed methods design, a symptom questionnaire that was preferred by experts and very positively assessed by patients was selected.

On the downside, the patient panel might be not representative of the entire advanced CKD population. First, patients participating in an online panel may be more health conscious, familiar with online questionnaires and involved in healthcare compared to those who do not participate (i.e. healthy responder bias). Second, most participants in the patient panel received a kidney transplant. However, the results and comments on the questionnaires of the transplant patients did not differ from those of the patients on dialysis or without RRT. Besides this, within the context of interest, patients will be followed over time, through different stages and treatments. Many patients receive (pre-)dialysis care prior to their transplantation, and thus, we do not expect that the inclusion of patients who received a kidney transplant affected the evaluation of the questionnaires.

With the method used, the focus was on the most important PROM property, namely the content validity of the symptom questionnaire. Additionally, the DSI showed good reliability: excellent internal consistency of the symptom burden score in this current study and good test-retest reliability in the development-study [34]. However, more research is needed to further explore the reliability and validity of this questionnaire [34]. Additionally, further research is needed to investigate if the DSI detects (clinically relevant) changes in symptom burden (i.e. responsiveness). Moreover, the smallest detectable change and the minimal important change need to be investigated for the interpretation of changes in symptom burden over time [53].

## Conclusion

In conclusion, the DSI was found to be valid and reliable, the most relevant, complete, and comprehensible symptom questionnaire currently available for routine assessment in patients with advanced CKD or ESKD. The use of PROMs could be of great added value to healthcare, both at the individual patient and national level. Feedback on results and involvement of healthcare providers may promote adaptation and implementation of PROMs into healthcare.

## Additional files

Additional file 1: Search string for systematic literature search for symptom questionnaires used in patients with chronic kidney disease. (DOCX 30 kb)

Additional file 2: **Table S2.** Unique symptoms identified from questionnaires and interviews with patients with chronic kidney disease, divided into ten symptom clusters. (DOCX 34 kb)

Additional file 3: **Table S3.** Comparison of two CKD-specific symptom questionnaires based on feedback of transplant and non-transplant patients. (DOCX 32 kb)

## Data Availability

The datasets used and/or analysed during the current study are available from the corresponding author on reasonable request.

## Abbreviations

CKD: Chronic Kidney Disease
COSMIN: COnsensus-based Standards for the selection of health Measurement INstruments
DSI: Dialysis Symptom Index
ESKD: End-Stage Kidney Disease
HRQOL: Health-Related Quality Of Life
IPOS-Renal: Palliative Care Outcome Scale – Renal Version
NVN: Dutch Kidney Patients Association
PRO(s): Patient-Reported Outcome(s)
PROM(s): Patient-Reported Outcome Measure(s)
RRT: Renal Replacement Therapy
SD: Standard Deviation
