# Supplementary material for: Patient-reported outcome measures: selection of a valid questionnaire for routine symptom assessment in patients with advanced chronic kidney disease – a four-phase mixed methods study
Source: BMC Nephrol. 2019 Sep 2;20:344. doi: 10.1186/s12882-019-1521-9 (PMC6720373; doi:10.1186/s12882-019-1521-9)
Supplement: Supplementary file 1 — Search string for systematic literature search for symptom questionnaires used in patients with chronic kidney disease. (DOCX 30 kb) [file 12882_2019_1521_MOESM1_ESM.docx]

| **Additional file 1. Search string for systematic literature search for symptom questionnaires used in patients with chronic kidney disease.** |
| --- |
| **((("Chronic Kidney Disease"[ti] OR "Chronic Kidney Diseases"[ti] OR "Chronic Renal Disease"[ti] OR "Chronic Renal Diseases"[ti] OR "CKD"[ti] OR "End-Stage Renal Disease"[ti] OR "End-Stage Renal Diseases"[ti] OR "ESRD"[ti] OR "End-Stage Kidney Disease"[ti] OR "End-Stage Kidney Diseases"[ti] OR "Advanced Renal Disease"[ti] OR "Advanced Kidney Disease"[ti] OR "Renal Insufficiency, Chronic"[majr] OR "Chronic Renal Insufficiency"[ti] OR "Chronic Renal Failure"[ti] OR "Chronic Kidney Failure"[ti] OR (("Kidney Diseases"[majr] OR "kidney disease"[ti] OR "renal disease"[ti] OR "kidney diseases"[ti] OR "renal diseases"[ti] OR "kidney failure"[ti] OR "renal failure"[ti] OR "renal insufficiency"[ti] OR "kidney insufficiency"[ti]) AND ("Chronic Disease"[majr] OR "chronic"[ti] OR chronic*[ti])) OR (("pre-dialysis"[ti] OR pre-dialy*[ti] OR "predialysis"[ti] OR predial*[ti] OR "chronic renal"[ti] OR "chronic kidney"[ti] OR "Renal Insufficiency, Chronic"[majr] OR "Kidney Failure, Chronic"[majr] OR "end stage renal"[ti] OR "end stage kidney"[ti]) AND ("3"[ti] OR "4"[ti] OR "5"[ti] OR "three"[ti] OR "four"[ti] OR "five"[ti] OR "iii"[ti] OR "iv"[ti] OR "v"[ti]) AND ("stage"[ti] OR "stages"[ti] OR "late"[ti])) OR "Renal Replacement Therapy"[majr] OR "Renal Replacement Therapy"[ti] OR "RRT"[ti] OR "hemodialysis"[ti] OR "haemodialysis"[ti] OR "peritoneal dialysis"[ti] OR "Kidney Transplantation"[ti] OR "Renal Transplantation"[ti] OR Kidney Transplant*[ti] OR Renal Transplant*[ti] OR "Dialysis"[majr] OR "Dialysis"[ti] OR "hemodiafiltration"[ti] OR "haemodiafiltration"[ti])**  **AND ("Signs and Symptoms"[Mesh:noexp] OR "Signs and Symptoms"[majr] OR "Symptom"[ti] OR "symptoms"[ti] OR "Symptom burden"[tw] OR symptom*[ti])**  **AND ("Surveys and Questionnaires"[Mesh:noexp] OR "Patient Reported Outcome Measures"[Mesh] OR "Questionnaire"[tw] OR "Questionnaires"[tw] OR Questionnair*[tw] OR "Patient-Reported Outcome Measure"[tw] OR "Patient-Reported Outcome Measures"[tw] OR "PROMs"[tw] OR "PROM"[tw] OR "Self Report"[tw] OR "assessment instrument"[tw] OR "assessment system"[tw] OR "assessment method"[tw] OR "assessment instruments"[tw] OR "assessment systems"[tw] OR "assessment methods"[tw] OR "Assessment Scale"[tw] OR "Assessment Scales"[tw] OR "instrument"[ti] OR "scale"[ti] OR "checklist"[ti] OR "score"[ti] OR "instruments"[ti] OR "scales"[ti] OR "checklists"[ti] OR "scores"[ti] OR "inventory"[ti] OR "inventories"[ti] OR "Symptom Burden Index"[tw]** **OR "symptom burden instrument"[tw] OR "symptom burden measures"[tw] OR "symptom burden score"[tw] OR "symptom burden scores"[tw])**  **AND (english[la] OR dutch[la])**  **NOT (("Adolescent"[mesh] OR Adolescen*[ti] OR "Child"[mesh] OR "child"[ti] OR "children"[ti] OR "girl"[ti] OR "girls"[ti] OR "boy"[ti] OR "boys"[ti]) NOT ("Adult"[mesh] OR "adult"[ti] OR "adults"[ti])))**  **OR**  **(("Chronic Kidney Disease"[ti] OR "Chronic Kidney Diseases"[ti] OR "Chronic Renal Disease"[ti] OR "Chronic Renal Diseases"[ti] OR "CKD"[ti] OR "End-Stage Renal Disease"[ti] OR "End-Stage Renal Diseases"[ti] OR "ESRD"[ti] OR "End-Stage Kidney Disease"[ti] OR "End-Stage Kidney Diseases"[ti] OR "Advanced Renal Disease"[ti] OR "Advanced Kidney Disease"[ti] OR "Renal Insufficiency, Chronic"[majr] OR "Chronic Renal Insufficiency"[ti] OR "Chronic Renal Failure"[ti] OR"Chronic Kidney Failure"[ti] OR (("Kidney Diseases"[majr] OR "kidney disease"[ti] OR "renal disease"[ti] OR "kidney diseases"[ti] OR "renal diseases"[ti] OR "kidney failure"[ti] OR "renal failure"[ti] OR "renal insufficiency"[ti] OR "kidney insufficiency"[ti]) AND ("Chronic Disease"[majr] OR "chronic"[ti] OR chronic*[ti])) OR (("pre-dialysis"[ti] OR pre-dialy*[ti] OR "predialysis"[ti] OR predial*[ti] OR "chronic renal"[ti] OR "chronic kidney"[ti] OR "Renal Insufficiency, Chronic"[majr] OR "Kidney Failure, Chronic"[majr] OR "end stage renal"[ti] OR "end stage kidney"[ti]) AND ("3"[ti] OR "4"[ti] OR "5"[ti] OR "three"[ti] OR "four"[ti] OR "five"[ti] OR "iii"[ti] OR "iv"[ti] OR "v"[ti]) AND ("stage"[ti] OR "stages"[ti] OR "late"[ti])) OR "Renal Replacement Therapy"[majr] OR "Renal Replacement Therapy"[ti] OR "RRT"[ti] OR "hemodialysis"[ti] OR "haemodialysis"[ti] OR "peritoneal dialysis"[ti] OR "Kidney Transplantation"[ti] OR "Renal Transplantation"[ti] OR Kidney Transplant*[ti] OR Renal Transplant*[ti] OR "Dialysis"[majr] OR "Dialysis"[ti] OR "hemodiafiltration"[ti] OR "haemodiafiltration"[ti])**  **AND ("Signs and Symptoms"[Mesh:noexp] OR "Signs and Symptoms"[majr] OR "Symptom"[tw] OR "symptoms"[tw] OR "Symptom burden"[tw] OR symptom*[tw])**  **AND ("Surveys and Questionnaires"[majr:noexp] OR "Patient Reported Outcome Measures"[majr] OR "Questionnaire"[ti] OR "Questionnaires"[ti] OR Questionnair*[ti] OR "Patient-Reported Outcome Measure"[ti] OR "Patient-Reported Outcome Measures"[ti] OR "PROMs"[ti] OR "PROM"[ti] OR "Self Report"[ti] OR "assessment instrument"[ti] OR "assessment system"[ti] OR "assessment method"[ti] OR "assessment instruments"[ti] OR "assessment systems"[ti] OR "assessment methods"[ti] OR "Assessment Scale"[ti] OR "Assessment Scales"[ti] OR "instrument"[ti] OR "scale"[ti] OR "checklist"[ti] OR "score"[ti] OR "instruments"[ti] OR "scales"[ti] OR "checklists"[ti] OR "scores"[ti] OR "inventory"[ti] OR "inventories"[ti] OR "Symptom Burden Index"[ti]** **OR "symptom burden instrument"[ti] OR "symptom burden measures"[ti] OR "symptom burden score"[ti] OR "symptom burden scores"[ti])**  **AND (english[la] OR dutch[la])**  **NOT (("Adolescent"[mesh] OR Adolescen*[ti] OR "Child"[mesh] OR "child"[ti] OR "children"[ti] OR "girl"[ti] OR "girls"[ti] OR "boy"[ti] OR "boys"[ti]) NOT ("Adult"[mesh] OR "adult"[ti] OR "adults"[ti]))))** |
