# Supplementary material for: Patient-reported outcome measures: selection of a valid questionnaire for routine symptom assessment in patients with advanced chronic kidney disease – a four-phase mixed methods study
Source: BMC Nephrol. 2019 Sep 2;20:344. doi: 10.1186/s12882-019-1521-9 (PMC6720373; doi:10.1186/s12882-019-1521-9)
Supplement: Supplementary file 2 — Table S2. Unique symptoms identified from questionnaires and interviews with patients with chronic kidney disease, divided into ten symptom clusters. (DOCX 34 kb) [file 12882_2019_1521_MOESM2_ESM.docx]

| **Additional file 2: Table S2. Unique symptoms identified from questionnaires and interviews with patients with chronic kidney disease, divided into ten symptom clusters.** | |
| --- | --- |
|  |  |
| **General symptoms** |  |
| Fatigue / feeling tired / lack of energy |  |
| Change in weight |  |
| Difficulty concentrating |  |
| Feeling sick |  |
| Pain (in general) |  |
| Changes in appearance |  |
|  |  |
| **Night’s rest** |  |
| Trouble falling asleep |  |
| Trouble staying asleep |  |
| Changes in amount of sleep |  |
| Drowsiness |  |
|  |  |
| **Gastroenterology** |  |
| Constipation |  |
| Nausea |  |
| Vomiting |  |
| Diarrhoea |  |
| Decreased appetite / lack of appetite |  |
| Feeling of fullness or bloating |  |
| Abdominal pain / stomach cramps |  |
| Heartburn |  |
| Stomach or bowel problems |  |
| Overeating / food cravings |  |
|  |  |
| **Cardiopulmonary** |  |
| Chest pain |  |
| Heart palpitations / arrhythmia |  |
| Easy bruising |  |
| Slow-healing sores |  |
| Shortness of breath / dyspnoea |  |
| Coughing |  |
| Wheezing |  |
| Swelling in legs / feet |  |
| Chest tightness |  |
| Nycturia |  |
|  |  |
| **Central nervous system** |  |
| Light-headedness or dizziness |  |
| Numbness in feet or hands |  |
| Tingling in feet or hands |  |
| Headache |  |
| Restless legs or difficulty keeping legs still |  |
| Shivering / hot or cold spells |  |
| Trembling |  |
| Trouble remembering things / memory loss |  |
| Sluggish / react slowly |  |
| Difficulty keeping attention |  |
| Inadequate / having to (double-)check what you do |  |
| Pain / burning / frequency of urination |  |
|  |  |
| **Musculoskeletal** |  |
| Muscle loss |  |
| Muscle cramps |  |
| Stiffening of joints |  |
| Bone or joint pain / pain in arms, legs or joints |  |
| Muscle soreness |  |
| Back pain |  |
| Muscle spasm |  |
| Muscle weakness |  |
| Poor mobility |  |
| Pelvic pain |  |
| Humps in muscles* |  |
|  |  |
| **Skin** |  |
| Dry skin |  |
| Itching / pruritus |  |
| Changes in skin |  |
| Loss of hair |  |
| Sweating |  |
|  |  |
| **Head/throat** |  |
| Dry mouth |  |
| Thirst |  |
| Change in taste |  |
| Pain when swallowing |  |
| Sore throat |  |
| Burning / sore eyes |  |
| Sore mouth |  |
| Hearing loss |  |
| Ringing in your ears |  |
| Impaired visual ability |  |
|  |  |
| **Psychosocial** |  |
| Feeling nervous |  |
| Feeling irritable |  |
| Feeling sad |  |
| Feeling anxious |  |
| Confusion |  |
| Worrying |  |
| Depressed mood |  |
| Restless |  |
| Tension / feeling tense or keyed up |  |
| Feeling blue |  |
| Feeling frustrated |  |
| Feeling angry |  |
| Feeling bored |  |
| Feeling lonely |  |
| Lack of vitality |  |
| Decreased motivation |  |
| Feel worn out |  |
| Difficulty making decisions |  |
| Feeling everything is an effort |  |
| Feeling of being trapped or caught |  |
| Feelings of guilt |  |
| Thoughts of ending your life |  |
| Difficulties with family life and social contacts |  |
| Feeling critical of others |  |
| Difficulties to trust others |  |
| Intrusive thoughts |  |
| Personality changes |  |
| Emotional swings |  |
| Despairing about the future |  |
| A lump in your throat |  |
|  |  |
| **Sex** |  |
| Decreased interest in sex |  |
| Difficulty becoming sexually aroused |  |
| Inability to relax and enjoy sex |  |
|  |  |
| *Symptom retrieved from videotaped interviews with patients with chronic kidney disease. |  |
