# Supplementary material for: Patient-reported outcome measures: selection of a valid questionnaire for routine symptom assessment in patients with advanced chronic kidney disease – a four-phase mixed methods study
Source: BMC Nephrol. 2019 Sep 2;20:344. doi: 10.1186/s12882-019-1521-9 (PMC6720373; doi:10.1186/s12882-019-1521-9)
Supplement: Supplementary file 3 — Table S3. Comparison of two CKD-specific symptom questionnaires based on feedback of transplant and non-transplant patients. (DOCX 32 kb) [file 12882_2019_1521_MOESM3_ESM.docx]

| **Additional file 3: Table S3. Comparison of two CKD-specific symptom questionnaires based on feedback of transplant and non-transplant patients.** | | | | | |
| --- | --- | --- | --- | --- | --- |
|  | **DSI (N=76)** | | **IPOS-Renal (N=75)** | | ***p*-value** |
|  | **Transplant (N=53)** | **Non-transplant (N=23)** | **Transplant (N=47)** | **Non-transplant (n=28)** |  |
|  |  |  |  |  |  |
| **Age** (years) | 60.4 (11.6) | 61.2 (14.6) | 59.7 (10.9) | 61.1 (9.7) | 0.941 |
| **Objective time to complete*** (minutes) | 5.0 (1.6) | 6.3 (1.4) | 7.4 (1.9) | 7.6 (1.6) | **0.001^a^** |
| **Subjective time to complete*** (minutes) | 3.0 (1.8) | 3.9 (1.7) | 5.0 (1.7) | 4.4 (1.5) | **<0.001^b^** |
| **Number of symptoms reported^** | 11.0 (6.7) | 14.4 (5.5) | 7.4 (4.1) | 9.0 (3.9) | **<0.001^c^** |
| **Additional 1-3 symptoms reported^#^** | 12 (22.6) | 9 (39.1) | 18 (38.1) | 7 (25.0) | 0.251 |
| **Burdensome of questionnaire** (yes) | 4 (7.5) | 0 (0.0) | 1 (2.2) | 1 (3.7) | 0.387 |
| **Appropriate frequency of submission** (times per year) | 2.7 (2.0) | 2.9 (1.6) | 2.4 (1.4) | 3.8 (2.9) | 0.072 |
| Values are shown in N (%) or mean (SD). | | | | | |
| ^a^ Post hoc tests: differences are statistically significant between DSI transplant and IPOS-Renal transplant, and between DSI transplant and IPOS-Renal non-transplant groups. ^b^ Post hoc tests: differences are statistically significant between DSI transplant and IPOS-Renal transplant, and between DSI transplant and IPOS-Renal non-transplant groups. ^c^ Post hoc tests: differences are statistically significant between DSI transplant and IPOS-Renal transplant, between DSI non-transplant and IPOS-Renal transplant, and between DSI non-transplant and IPOS-Renal non-transplant groups. | | | | | |
| *Objective time to complete was defined as the difference in minutes between the start and completion of the online questionnaire. Subjective time to complete is the time to complete estimated by the patient. Values shown as geometric mean (SD). | | | | | |
| ^The number of symptoms reported is based on the symptoms defined in the questionnaire and rated by the patient as bothering a little bit to very much (or affecting slightly to overwhelmingly). | | | | | |
| ^#^The number of patients reporting an additional 1 to 3 symptoms not mentioned in de questionnaire. | | | | | |
| CKD, Chronic Kidney Disease; PROMs, Patient Reported Outcome Measures; DSI, Dialysis Symptom Index; IPOS-Renal, Palliative Care Outcome Scale - Renal Version. | | | | | |
